# Soluble fibers from psyllium improve glycemic response and body weight among diabetes type 2 patients (randomized control trial)

**DOI:** 10.1186/s12937-016-0207-4

**Published:** 2016-10-12

**Authors:** Ayman S. Abutair, Ihab A. Naser, Amin T. Hamed

**Affiliations:** Department of Clinical Nutrition, Faculty of Pharmacy, Al-Azhar University Gaza, Gaza Strip, Palestine

**Keywords:** Diabetes, Fiber, Fasting blood sugar, Insulin, Body weight

## Abstract

**Background:**

Water-soluble dietary fibers intake may help control blood glucose and body weight.

**Objective:**

The objective of the study was to determine whether soluble fiber supplementation from psyllium improves glycemic control indicators and body weight in type 2 diabetic patients.

**Method:**

Forty type 2 diabetes patients, non-smoker, aged >35 years were stratified to different strata according to sex, age, body mass index (BMI) and fasting blood sugar level (FBS) and randomly assigned into two groups; The intervention group which consists of 20 participants was on soluble fiber (10.5 g daily), and the control group which consist of 20 participants continued on their regular diet for eight weeks duration.

**Results:**

After 8 weeks of intervention, soluble fiber supplementation showed significant reduction in the intervention group in BMI (*p* < 0.001) when compared with the control group. Moreover, water soluble fiber supplementation proven to improve FBS (163 to 119 mg/dl), HbA1c (8.5 to 7.5 %), insulin level (27.9 to 19.7 μIU/mL), C-peptide (5.8 to 3.8 ng/ml), HOMA.IR (11.3 to 5.8) and HOMA-β % (103 to 141 %).

**Conclusion:**

The reduction in glycemic response was enhanced by combining soluble fiber to the normal diet. Consumption of foods containing moderate amounts of these fibers may improve glucose metabolism and lipid profile in type 2 diabetes patients.

**Trial registration:**

Current Controlled Trials PHRC/HC/28/15.

## Background

Diabetes is the fastest growing chronic condition worldwide. According to Centers for Disease Control and Prevention (CDC), 9.3 % of the U.S. population have diabetes [1]. By 2035 the number of adults having diabetes will increase from 387 to 592 million worldwide [2]. It’s now well established that diabetes increases the chances for serious complication such as heart diseases and associated mortality [3]. Diet, exercise and behavioral approaches represent the key for management and prevention of diabetes [4, 5]. More important, consumption of dietary fibers were inversely related to type 2 diabetes and cardiovascular disease [6]. Researchers stressed that, consumption of more than 26 g a day had an 18 % lower risk of developing type 2 diabetes than those with the lowest intake [7]. Fiber is commonly classified as soluble, which dissolves in water or insoluble, which doesn’t dissolve [8]. The soluble fibers can dissolves in water to form a gel-like material. Soluble fiber is found in oats, peas, beans, apples, citrus fruits, carrots, barley and psyllium [8]. Interestingly, the solubility of soluble fiber refers to its ability to dissolve in water, forming viscous gels, bypasses the digestion of the small intestine [9], and slows absorption and digestion of carbohydrates [10]. Psyllium is a water-soluble fiber derived from the husks of ripe seeds from Plantago ovate [11], that may improves glycemic control [12–14], body weight [15] and improve bowel movement [16] in patients with type 2 diabetes. On the other hand, increased consumption of high glycemic index food was associated with insulin resistance and increased prevalence of metabolic syndrome in patients with type 2 diabetes [17]. The purpose of this study was to investigate the beneficial effects of adding soluble fiber to the normal diet for eight weeks of intervention among diabetes type 2 patients.

## Methods and material

### Study design and setting

This study utilized randomized controlled trial design, with newly identified Type 2 diabetes patients (maximum one year) who were on anti-diabetes medication. Aged more than 35 years of old, male and female and who attend private endocrine clinics in the Gaza Strip. Sample size was calculated using two mean formula, which indicated that 18 respondents in each subjects. However, to ensure power to reach desired statistical outcomes and allow for voluntarily withdrawal, number of subjects were increased to 20 in per group. The study included 20 male and 20 female patients. To insure equal distribution of cases, the subjects were stratified according to sex (male/female), age (35–45 years/45–50 years/50–55 years/55–60 years), BMI (25–29.99/30–34.99/35–39.99) and fasting glucose level (110–149 mg, 150–200 mg) into homogeneous strata, then the subjects were allocated randomly in to intervention and control group. The control group consists of 20 participants did not receive any food supplements throughout the intervention period and instead continued with their regular diets. While, the intervention group consists of 20 participants, and received soluble fiber 10.5 g daily for eight weeks of intervention.

### Intervention protocol

Soluble fiber was provided to the intervention group in this study, whereas the control group remained on their regular diet. Both groups were on their regular diet and lifestyle. The intervention protocol was designed in a way that 7.0 g of psyllium was given to the intervention group 15 min before lunch and 3.5 g of psyllium 15 min before dinner with 150 ml of water with each dose. To avoid choking while consuming the psyllium supplements. Respondents instructed to consume psyllium with an adequate amount of water (first melt the fiber dose well in 100 ml of water and drink it, then drink additional 50 ml of water). To insure subjects compliance to the intervention program, subjects were contacted by phone 3 times weekly and they were instructed to fill up the compliance checklists to record the daily consumption of the soluble fiber doses, these checklists were collected weekly.

### Dietary stabilization phase

During a dietary stabilization phase, subjects in both groups were instructed to follow a prescribed diet plan for one week for diabetes, to stabilize the serum glucose level (≤30 % of total energy as fat,≤10 % of energy as saturated fat, and ≥55 % of energy as carbohydrate- focused on complex carbohydrate). Dietary stabilization was not the major focus of this intervention and the main goal of dietary instruction was to encourage subjects to maintain their same dietary patterns throughout the stabilization phase. Also, we have prepared a diet program for diabetes patients for one week, and calories was calculated based on body weight for each participant (25 Kcal multiplied by body weight Kg).

### Measurements

Anthropometric measurements and biochemical indicators were taken at the baseline, and at the end of the trial. Weight was taken in kilograms and grams. Also, respondents were weighted pre and post intervention with same minimal clothing, without shoes or other heavy accessories such as mobile phones and wallets. Heights measurements were taken using calibrated Stadiometer. Respondents stand with bare feet that are kept together and the head was level with a horizontal Frankfurt plane below an imaginary line from the lower border of the eye orbit to the auditory meatus [18]. Heights were measured in centimeters and millimeters. In addition, the commonly accepted BMI ranges are underweight: under 18.5, normal weight: from 18.5 to 24.9, overweight: from 25 to 29.9, obese: over 30. Furthermore, waist circumference was measured at the midpoint between the lower margin of the last palpable rib and the top of the iliac crest, using a stretch resistant tape [19]. According to the WHO, waist circumference more than 102 cm for adult male and more than 88 cm for adult non-pregnant female indicate risk for obesity and its health complications risks [19]. Also, hip circumference is measured in a similar manner to waist circumferences, with the tape being passed around the hips at the widest circumference of the buttocks and the tape measure be kept level and parallel to the floor whilst the measurement is made. The average of three measurements was recorded. Waist to hip ratio (WHR) is the ratio of the circumference of the waist to that of the hips. This is calculated as waist measurement divided by hip measurement (W/H). WHR is used as a measurement of obesity, which in turn is a possible indicator of other more serious health conditions. The WHO states that abdominal obesity is defined as a WHR above 0.90 for males and above 0.85 for females [19]. The Measured biochemical parameters include insulin level, FBS, hemoglobin A1c, C-peptide. HOMA-IR and HOMA-B% were calculated in order to predict insulin sensitivity and Beta cell function according to the following formulas: HOMA-IR = (glucose mg * insulin level)/405, and HOMA-B% = (360* insulin level)/(glucose mg – 63) [20].

### Kits and devices

The device used for F.B.S. analysis was (Mindry semi-auto chemistry, model BA-88A, S.N WR-04002031) and the kit that was used in this device was (Bio Med- Glucose L.S, normal level in the plasma 60–110 mg/dl, and sensitivity: 0.4 mg/dl). The device used for HbA1c analysis was (ClOVER A1c analyzer, model IGM-0023, S.N GWLHCLD0056) and the kit used was (CLOVER A1c test cartridge and used with ClOVER A1c analyzer only, less than 7 % is the treatment goal). The device used for insulin and C-peptide analysis was (Mindry micoplate reader, model MR-691A, S.N. 4100566) and the kits used for Insulin was (DRG Insulin ELISA EIA-2935, normal value 2–25 μIU/mL, sensitivity: 1.76 μIU/mL) and for C-Peptide was (DRG C-Peptide ELISA EIA-1293, normal value: 0.5-3.2 ng/ml, sensitivity: 0.064 ng/ml).

### Data analysis

Following data collection, all data was reviewed before being entered into the study database. Data were entered into an SPSS (Statistical Package for Social Sciences) version 18.0 for Windows. The data treatment involved cleaning and organizing the data for analysis, which included numbering the results, codifying the data, entering the data into the computer, checking the data for accuracy. The data analysis was divided into two steps: descriptive statistics and analysis of variance. The data are presented in tables. The quantitative data are represented in the form of proportions (%) and of means with standard deviations or medians. All measurements and indicators of the two groups were compared at the baseline, and after eight weeks of intervention to evaluate the impact of the intervention. To compare the changes in the glycemic control indicators and anthropometric measurements one-way repeated measure ANOVA was used. The level of significance was set at 0.05.

## Results

Out of 40 subjects four respondents withdrew during the study period, and 36 respondents have completed the trial from the beginning to the end of the study successfully. None of the remaining participants were removed from the entire database, and no missing data was observed of all important variables.

### Anthropometric characteristics for both groups at baseline level

Table 1 presents the anthropometric characteristics of the respondents for both groups. The mean weight for respondents was 89 kg SD (13.91), the mean height was 1.7 m SD (0.11) and the mean BMI was 31.7 (kg/m^2^) SD (2.71), the majority of the respondents 25 (69.4 %) were obese and ranged from class I to class II. The mean waist circumferences was 106.8 (7.30) cm, and the results showed that the majority of male respondents 17 (94.4 %) waist circumference were more than normal cut of points 102 cm for adult male. All female respondents waist circumference were more than normal cut of points 88 cm for adult female. The mean hip circumferences for respondents was 108.8 (7.79) cm. The WHR mean of all respondents was 0.99 (0.09).

**Table 1:** Anthropometric characteristics of all respondents

Variables	Total	
	*n* (%)	Mean (SD)
Weight		89 (13.91)
Height		1.7 (0.11)
BMI		31.7 (2.71)
Normal	0 (0.00)	
Overweight	11 (30.6)	
Obese	25 (69.4)	
Waist circumferences		106.8 (7.30)
More than 102 cm (male)	17 (94.4)	
Less than 102 cm (male)	1 (5.6)	
More than 88 cm (female)	18 (100)	
Less than 88 cm (female)	0 (0.00)	
Hip circumferences		108.8 (7.79)
Waist to Hip Ratio ^a^		0.99 (0.09)
0.95 or less low risk (male)	4 (22.2)	
0.96–1.0 moderate risk (male)	2 (11.1)	
More than 1.0 high risk (male)	12 (66.7)	
0.80 or less low risk (female)	0 (0.00)	
0.81–0.85 Moderate risk (female)	2 (11.1)	
More than 0.85 high risk (female)	16 (88.9)	

^a^ Health Risk Based Solely on waist to hip ratio (WHR) for male (low risk 0.95 or below, moderate risk 0.96–1 and high risk more than 1, and for female (low risk 0.95 or below, moderate risk 0.96–1 and high risk more than 1

### Changes in the anthropometric measurements 

After 8 weeks of intervention, significant changes were observed in the majority of anthropometric measurements in either group (Table 2). In intervention group, the weight, BMI, waist circumference and hip circumference were significantly decreased (weight (2.7 kg, *p* < 0.001), BMI (0.98 kg/m^2^, *p* < 0.001), waist circumference (2.6 cm, *p* < 0.001), and hip-circumference (2.5 cm, *p* < 0.001)). The WHR shows some reduction about 0.0019, in which this reduction was not significant. Psyllium supplementation showed significant improvement on anthropometrics as compared with the control group by repeated test for treatment (time x treatment interaction).

**Table 2:** Differences in anthropometrics in the control and intervention groups

Variables	Control (*n* = 18)		Intervention (*n* = 18)		*P* value ^a^
	Pre	Post	Pre	Post	
Weight (kg)	87.3 (13.45)^b^	88.1 (13.379)**	91.7 (14.42)	88.8 (14.78)***	<0.001
BMI (kg/m^2^)	31.5 (2.66)	31.8 (2.66)**	31.8 (2.82)	30.9 (2.94)***	<0.001
W.C. (cm)	107.5 (7.07)	107.9 (6.77)	106.2 (7.66)	103.5 (7.65)***	<0.001
Hip.C. (cm)	107.8 (7.84)	108.3 (7.87)*	109.9 (7.82)	107.3 (7.96)***	<0.001
WHR	1.00 (0.100)	1.00 (0.100)	0.97 (0.073)	0.97 (0.072)	0.912

^a^ Repeated Measure ANOVA between Control and intervention groups

^b^ Mean (Standard Deviation)

The level of significance is < 0.05

Asterisk = significantly different by paired *t*-test between baseline and 8th week in the same intervention group, *p < 0.05, **p < 0.01, *** *p* < 0.001

### Glycemic control indicators characteristics for both groups at baseline level

Table 3 presents the glycemic control characteristics of the respondents for both groups. The mean FBS was 160 mg/dl, and all the respondents were above normal cut off point which is 110 mg/dl. The mean of HbA1c was 8.5 (0.654) and the majority of respondents 26 (72.2 %) were above normal cut off point which is from 6 to 8 %, and the mean insulin level was 24.8 μIU/mL, and 50 % of respondents were in normal reference range (2–25 μIU/mL), and 50 % were higher than the normal reference range, In addition, the mean C-peptide level was 5.5 ng/ml, and the majority of respondents 35 (97.2 %) were above the normal reference range which is 0.5–3.2 ng/ml. Moreover, the mean HOMA-IR and HOMA-B% were 9.9 and 94.2 respectively.

**Table 3:** Glycemic control indicators characteristics of all respondents

Variables	Total	
	*n* (%)	Mean (SD)
Fasting blood sugar		160 (18.33)
Normal range	1 (1.7)	
110-149 mg/dl	14 (38.9)	
150–200 mg/dl	22 (61.1)	
HbA1c		8.5 (0.654)
Within normal level %	10 (27.8)	
More than 8 %	26 (72.2)	
Insulin level		24.8 (5.916)
Within normal range	18 (50.00)	
More than 25	18 (50.00)	
C-peptide		5.5 (1.51)
Within normal range	1 (2.8)	
More than 5	35 (97.2)	
HOMA-IR		9.9 (2.824)
HOMA-B%		94.2 (24.375)

### Changes in the glycemic control indicators

Changes of glycemic control indicators after 8 weeks of intervention are shown in Table 4. In which FBS, HbA1c, insulin level, C-peptide and HOMA.IR. HOMA.B% were improved significantly in intervention group (43.6 mg/dl *p* < 0.001, 0,9 % *p* = 0.013, 8.3 μIU/mL *p* < 0.001, 2 ng/ml *p* < 0.001, 5.5 *p* < 0.001, respectively) after 8 weeks of intervention. As a result, the supplementation of soluble fiber improved the majority of glycemic control indicators significantly in the intervention group as compared with the control group by repeated test for treatment (time x treatment interaction).

**Table 4:** Differences in glycemic control indicators in the control and intervention groups

Variables	Control (*n* = 18)		Intervention (*n* = 18)		*P* value ^a^
	Pre	Post	Pre	Post	
FBS (mg/dl)	156 (18.28)^b^	151 (11.65)	163 (18.26)	120 (16.48)***	<0.001
HbA1c (%)	8.5 (0.62)	8.5 (0.56)	8.5 (0.69)	7.5 (0.54)***	<0.001
Insulin (μIU/mL)	21.8 (5.39)	24.9 (5.13)*	27.9 (4.81)	19.7 (2.90)***	<0.001
C-peptide (ng/ml)	5.2 (1.63)	6.3 (1.26)***	5.8 (1.35)	3.8 (0.91)***	<0.001
HOMA.IR	8.5 (2.62)	9.3 (2.15)	11.3 (2.34)	5.8 (1.12)***	<0.001
HOMA.B%	85.5 (21.40)	102.5 (21.77)*	102.9 (24.59)	140.7 (60.89)**	0.239

^a^ Repeated Measure ANOVA between Control and intervention groups

^b^ Mean (Standard Deviation)

The level of significance is < 0.05

Asterisk = significantly different by paired *t*-test between baseline and 8th week in the same intervention group, *p < 0.05, **p < 0.01, *** *p* < 0.001

## Discussion

### Anthropometric changes

At base line level, the mean BMI was 31.7 (2.71) (kg/m^2^), while the majority of the respondents 25 (69.4 %) were obese and ranged from class I to class II. The majority of male respondents 17 (94.4 %) waist circumferences were more than normal cut of points [21], and all of the female respondents 18 (100 %) waist circumferences were more than normal cut of points [21]. This study indicated that, the inclusion of 10.5 g of psyllium with daily diet decreased (weight (from 91.7 to 88.8 kg, *p* < 0.001), BMI (from 31.8 to 30.9 kg/m^2^, *p* < 0.001), waist circumference (from 106.2 to 107.3 cm, *p* < 0.001), hip circumference (from 109.9 to 107.3 cm, *p* < 0.001). Unfortunately, results showed significant increment in weight, BMI and waist circumference of the respondents in the control group. Our results are consistent with those of *pal* et al. study in which the body weight and other anthropometric measurements were significantly improved [22]. This changes in anthropometric measurements might be achieved by stimulation of satiety hormones production that’s enhancing satiety [23], Another study confirmed that, increase in fiber intake with regular diet has resulted desirable outcomes such as; increased satiety, reduced hunger, decreased energy intake, increase on chewing time and weight loss [24]. Moreover, fiber itself less energy dense if a large volume is consumed. Fiber-rich food is processed more slowly than other food types [25]. Increased consumption of soluble dietary fiber lead to increase the viscosity in the gastrointestinal tract, improving post meal satiety and decreased subsequent hunger [26–28]. More importantly, satiety can be reached by different proposed mechanisms which include; gastric distension [26], slower absorption of macronutrients resulting in a reduction in postprandial glycemia [29], and enhanced effects of hunger-related hormones such as cholecystokinin, glucagon-like peptide-1 and peptide YY [30]. However, not all studies indicated significant effects of dietry fibers in satiety and body weight regulation [24, 27, 31, 32].

### Glycemic control indicators changes

The magnitude of glycemic control indicators reduction seen in this study were seen in other clinical trials. *Feinglos* et al., in his clinical trial noted that 3.4 g dose and 6.8 g dose of psyllium significantly lowered HbA1c and FBS [33]. Another study concluded that, a psyllium dose of 5.1 g/day significantly reduced FBS and HbA1c [34]. Likewise, in the current study all glycemic control indicators were significantly improved when these changes compared FBS, HbA1c, insulin and C-peptide *p* < 0.001, *p* < 0.001, *p* < 0.001, *p* = 0.001, respectively). Interestingly, these positive results were achieved due to soluble fiber effect in reducing glucose absorption about 12.2 % and as therapeutic agent for other metabolic control [35], while regular intake of soluble fibers, particularly may have a protective role for the presence of metabolic syndrome of patients with type 2 DM [13]. Though, psyllium as an example of viscose functional water soluble fiber which may delay intestinal transit time and leading to feel fullness [36], retarding the entry of glucose into the bloodstream and lessening the postprandial rise in blood sugar [37]. It may lessen insulin requirements [38]. Soluble fiber may produce a slower and longer-lasting release and absorption of macronutrients due to increased intraluminal viscosity [26, 39]. In the intestine, the gel-like material that formed by soluble fiber traps nutrients inside its gel and slows down considerably while passing through the digestive tract. Inside the gel, nutrients are protected from the action of digestive enzymes and less likely to reach the wall of the intestines for absorption [40]. This lowers the sharp rise of blood sugar after meal, and improves the sensitivity of the cells to the action of insulin [40]. In addition, Water soluble fiber thickens the unstirred water layer covering the surface of the intestines, which make the nutrients more resistant to cross this layer and to diffuse into the body [41]. Also, it has been reported that, soluble fiber decreased postprandial glucose and insulin responses and influenced concomitant gastrointestinal (GI) peptide responses, especially ghrelin and Peptide YY release [39]. Another clinical trial showed that psyllium improve FBS, insulin, HOMA Index, HbA1c significantly [42]. Another randomized crossover study, demonstrated that high intake of dietary fiber, particularly of the soluble type, improves glycemic control, decreases hyperinsulinemia and lowers plasma lipid concentrations in patients with type 2 diabetes [43]. In addition, the use of HOMA for assessment of β-cell function and insulin sensitivity which can detect the pathophysiology in those with abnormal glucose tolerance, thus HOMA is useful analysis tool of the treatment [44]. Consequently, recent studies among hyperglycemic individuals with no prior diagnosis of diabetes mellitus found to improve insulin sensitivity and other important metabolic controls [45–49]. This is most likely due to the viscosity of soluble fibers inside the gastrointestinal tract [29].

## Conclusion

It seems that soluble fiber from psyllium deserves attention as a potential natural dietary supplements for use in nutritional rehabilitation of type 2 diabetic patients, as it is inexpensive and shows positive results within a short span of time.

## Abbreviations

BMI: Body mass index
CDC: Centers for disease control and prevention
FBS: Fasting blood sugar
HbA1c: Hemoglobin A1c
RCT: Randomized control trial
WHO: World Health Organization
WHR: Waist to hip ratio
